# Crystal structures of an Extracytoplasmic Solute Receptor from a TRAP transporter in its open and closed forms reveal a helix-swapped dimer requiring a cation for α-keto acid binding

**DOI:** 10.1186/1472-6807-7-11

**Published:** 2007-03-15

**Authors:** Sophie Gonin, Pascal Arnoux, Bénédicte Pierru, Jérôme Lavergne, Béatrice Alonso, Monique Sabaty, David Pignol

**Affiliations:** 1 CEA/Cadarache, DSV/DEVM, Laboratoire de Bioénergétique Cellulaire, 13108 St Paul lez Durance Cedex, France; 2 CEA/Valrhô, DSV/DIEP/SBTN, 30207 Bagnols-sur-Cèze, France

## Abstract

**Background:**

The import of solutes into the bacterial cytoplasm involves several types of membrane transporters, which may be driven by ATP hydrolysis (ABC transporters) or by an ion or H^+ ^electrochemical membrane potential, as in the tripartite ATP-independent periplasmic system (TRAP). In both the ABC and TRAP systems, a specific periplasmic protein from the ESR family (Extracytoplasmic Solute Receptors) is often involved for the recruitment of the solute and its presentation to the membrane complex. In *Rhodobacter sphaeroides*, TakP (previously named SmoM) is an ESR from a TRAP transporter and binds α-keto acids *in vitro*.

**Results:**

We describe the high-resolution crystal structures of TakP in its unliganded form and as a complex with sodium-pyruvate. The results show a limited "Venus flytrap" conformational change induced by substrate binding. In the liganded structure, a cation (most probably a sodium ion) is present and plays a key role in the association of the pyruvate to the protein. The structure of the binding pocket gives a rationale for the relative affinities of various ligands that were tested from a fluorescence assay. The protein appears to be dimeric in solution and in the crystals, with a helix-swapping structure largely participating in the dimer formation. A 30 Å-long water channel buried at the dimer interface connects the two ligand binding cavities of the dimer.

**Conclusion:**

The concerted recruitment by TakP of the substrate group with a cation could represent a first step in the coupled transport of both partners, providing the driving force for solute import. Furthermore, the unexpected dimeric structure of TakP suggests a molecular mechanism of solute uptake by the dimeric ESR via a channel that connects the binding sites of the two monomers.

## Background

Transport systems are required in all organisms to facilitate movement of nutrients and other solutes across biological membranes. In prokaryotes, several classes of transport systems have been defined on the basis of their subunit composition and mode of energization [1]. The well-characterized ATP-binding cassette superfamily (ABC) represents one of the largest families of solute-specific transporters. In the ABC system, the driving force for solute transport across the membrane subunits is derived from ATP hydrolysis. In bacteria, solute uptake often requires the presentation of substrate by a high affinity Extracytoplasmic Solute Receptor (ESR, also called S- or P-BP for Solute or Periplasmic Binding Protein). The three dimensional structures of numerous ESRs specific for a wide range of substrates have been determined and, despite lack of sequence similarity, all were found to adopt a similar ternary fold [2,3] where the substrate binding site is located at the interface of two α/β domains connected by a hinge. The transport cycle begins with substrate binding to the ESR, inducing a conformational change to a "closed form" whereby the solvent is excluded from the substrate (hence the model denomination as a "Venus flytrap"). The docking of the loaded ESR to the ABC complex triggers a conformational change of the latter, which induces the binding of ATP and its hydrolysis by the Nucleotide Binding Domain (NBD) [4]. The ESRs thus play a key role in both the recruitment of the specific substrate and the control of ATP hydrolysis by the NBD.

The requirement for solute recognition by a periplasmic subunit prior to its translocation is not specific to ABCs since ESRs are also found in ATP-independent secondary transporters, the so-called Tripartite ATP-independent Periplasmic transporters (TRAP). In TRAP systems, the periplasmic ESR (often called the P subunit) is associated with two membrane components: a large transmembrane subunit involved in the translocation process (the M subunit) and a smaller membrane component of unknown function (the Q subunit). TRAP transporters lack the sequence signature characteristic of NBD, and biochemical evidences suggests that their driving force does not come from ATP but rather from the free energy stored in an electrochemical ion gradient across the cytoplasmic membrane [5]. The molecular mechanisms encompassing *e.g*. the recognition of the solute-ESR complex and the coupling of the transport to the ion gradient remain unknown.

The TRAP family is widespread in prokaryotes, as predicted from sequence analysis of bacterial genomes [6]. However, the physiological role of few of them has been elucidated since ligands for ESRs of TRAP transporters have only been evidenced for C4-dicarboxylate [7], ectoine [8], glutamate [9], xylulose [10], and sialic acid [11]. The best characterized TRAP transporters at functional and molecular levels are the high-affinity C4-dicarboxylate transport system (dctPQM) from *Rhodobacter capsulatus *[5,12] and the sialic acid transporter (SiaPQM) from *Haemophilus influenzae *[11]. In the latter, the structure of the periplasmic subunit (SiaP) was solved very recently at high resolution, revealing, among others, an overall topology similar to ABC ESR proteins [13].

In this study, we have focussed on the structural characterization of SmoM, a member of the DctP family. The *smoM *gene was initially annotated as coding for a sorbitol/mannitol binding protein on the basis of its position in the genome, close to the *smo *operon encoding known sorbitol/mannitol catabolic genes [14]. There is now clear evidence that SmoM does not participate in sorbitol or mannitol transport. First, the gene *smoM *is more than 500 bp away from the *smo *operon. Second, two genes homologous to *dctQ *and *dctM *are located in the region immediately downstream of *SmoM*, forming a putative functional TRAP transporter. Finally, purified proteins from the SmoM family neither bind sorbitol nor mannitol but display a specificity for α-keto acid complexes ([15] and this study), consistent with the suggested role of this transporter for supplying intermediates in the synthesis of valine and isoleucine. We thus propose to rename SmoM as TakP (TRAP transporter alpha-keto acid binding P subunit) and, by the same token, the associated membrane proteins as TakQ and TakM (the small and large integral membrane proteins, respectively).

In this paper, we present the high resolution structures of TakP in its unliganded form and complexed with sodium-pyruvate. This study reveals a key role for an ion in the attachment mode of the substrate, as well as an unexpected dimerization largely mediated by a helix swapping. The molecular mechanism of solute uptake is discussed in the light of these unique structural findings.

## Results

### TakP, a secondary transporter of α-keto acids

We became initially interested in the study of TakP when we found that a *Rhodobacter sphaeroides *mutant carrying a single Tn5 insertion in *takP *displayed a higher resistance to selenite [16]. Recently, TakP from *R. capsulatus *was shown to bind monocarboxylic 2-oxoacid anions *in vitro *[15]. We carried out a phenotypic analysis of the *takP *mutant to determine the most physiologically relevant substrate of the Tak transporter. However, no phenotypic difference could be characterized when comparing mutant and parent strains cultivated in minimal media supplemented with various α-keto acids (not shown). This suggests the presence of another import system *in vivo *or a non essential role for this ESR in the transport process.

We overexpressed and purified TakP from *Rhodobacter sphaeroides *and confirmed its ability to bind a range of α-keto acids. A general feature of ESRs is that substrate binding is accompanied by a diminished fluorescence from some tryptophan residue(s) as a result of the conformational changes induced by the binding. Indeed, we found that about 30% of the tryptophan fluorescence emitted by TakP became quenched when adding a saturating concentration of substrate. This was accompanied by a shift of the emission peak: the difference spectrum between the unliganded and liganded (quenched) protein has its maximum around 345 nm, whereas the bulk fluorescence from the unliganded form peaks at 335 nm. These features suggest that the tryptophans which become quenched in the liganded configuration represent a more solvent-exposed fraction of the protein tryptophans in the unliganded structure. There are 10 Trp residues in the TakP sequence. From the structural information describe below, it turned out that one of these Trp residues (Trp 215) is directly interacting with the ligand when present, so that its fluorescence may be strongly quenched as a result. Two others undergo a significant displacement during the open/closed transition, which may also affect their fluorescence properties. Clearly, the observed amplitude of the fluorescence quenching caused by ligand binding (~30%) implies that these sensitive residues have for some reason a larger relative contribution to the fluorescence emission than the other tryptophans of the protein.

**Figure 1:**
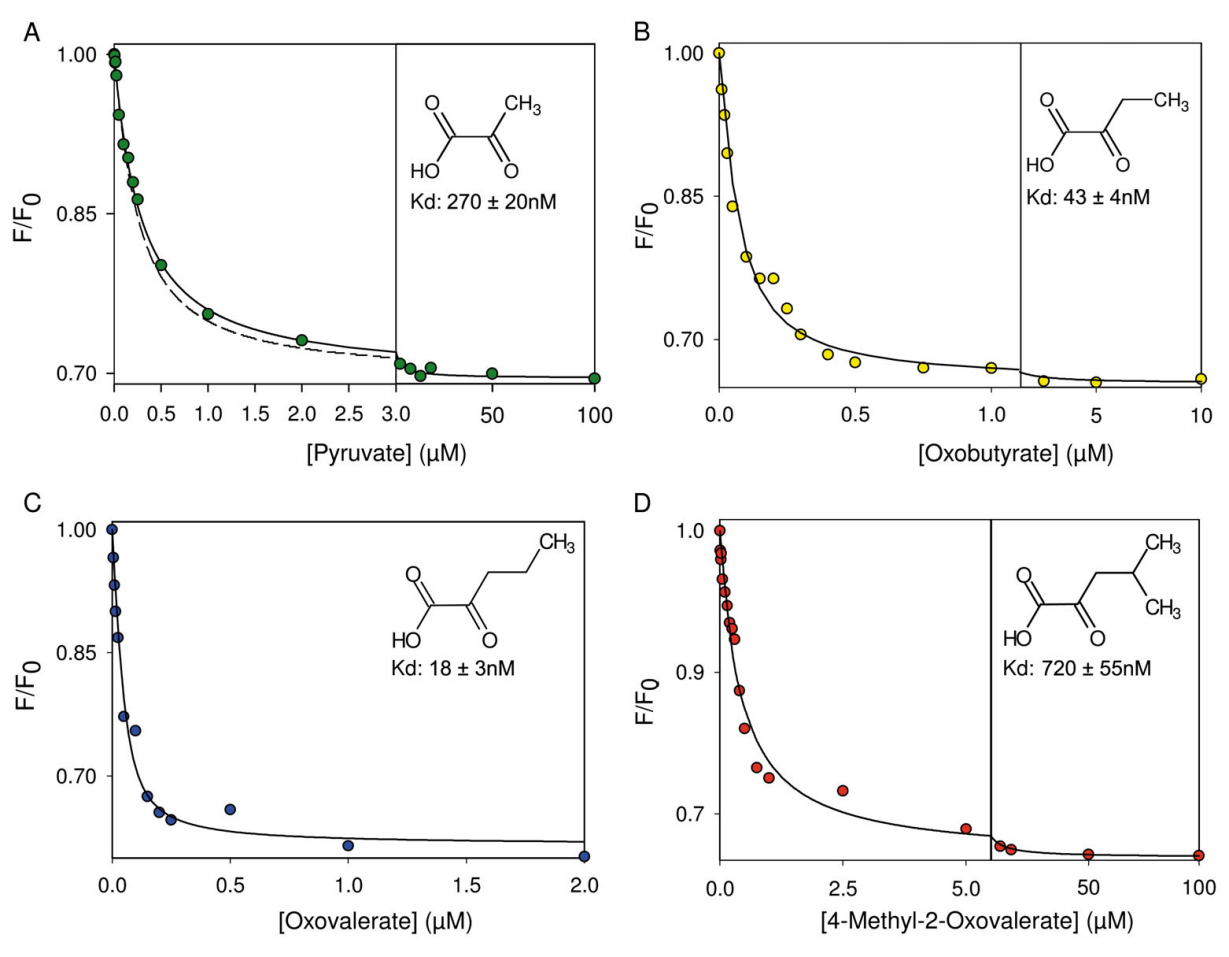
**Binding of 2-keto acids to TakP**. **A: **The amplitude of the integrated fluorescence band (normalized to its extent in the absence of ligand) was plotted as a function of the concentration of added pyruvate. The concentration of binding sites (monomeric unit) was 50 nM. The solid line is a fit with the equation given in the text, yielding K_*d *_≈ 270 nM. The dashed line is a numerical simulation of a (slightly) cooperative model, assuming that the first binding event occurs with K_*d*_(1) = 270 nM and the second one with Kd(2) = 0.75 × Kd(1). When fitted by a Hill equation, this model corresponds to a Hill number n ≈ 1.1. Any larger cooperativity would increase the sigmoidal character of the binding curve and could not be consistent with the data. **B-D: **Fluorescence amplitude change, molecular structure and dissociation constant obtained using oxobutyrate (B), oxovalerate (C) and 4-Methyl-2-Oxovalerate (D).

The fluorescence quenching was used to titrate the binding of various ligands, as shown in Figure 1. Because of the high binding affinity of these, we had to use a sufficiently low protein concentration to obtain an accurate determination of the dissociation constant, *K*_*d*_. If the protein concentration is below the *K*_*d*_, allowing the presence of a significant fraction of free substrate during the titration, the *K*_*d *_can be determined from a fit of the equation:

F/F0=1−ΔF×[PL]=1−ΔF×0.5(Kd+[P0]+[L0]−(Kd+[P0]+[L0])2−4[P0][L0])
 MathType@MTEF@5@5@+=feaafiart1ev1aaatCvAUfKttLearuWrP9MDH5MBPbIqV92AaeXatLxBI9gBaebbnrfifHhDYfgasaacH8akY=wiFfYdH8Gipec8Eeeu0xXdbba9frFj0=OqFfea0dXdd9vqai=hGuQ8kuc9pgc9s8qqaq=dirpe0xb9q8qiLsFr0=vr0=vr0dc8meaabaqaciaacaGaaeqabaqabeGadaaakeaacqWGgbGrcqGGVaWlcqWGgbGrdaWgaaWcbaGaeGimaadabeaakiabg2da9iabigdaXiabgkHiTiabfs5aejabdAeagjabgEna0kabcUfaBjabdcfaqjabdYeamjabc2faDjabg2da9iabigdaXiabgkHiTiabfs5aejabdAeagjabgEna0kabicdaWiabc6caUiabiwda1maabmaabaGaem4saS0aaSbaaSqaaiabdsgaKbqabaGccqGHRaWkcqGGBbWwcqWGqbaudaWgaaWcbaGaeGimaadabeaakiabc2faDjabgUcaRiabcUfaBjabdYeamnaaBaaaleaacqaIWaamaeqaaOGaeiyxa0LaeyOeI0YaaOaaaeaacqGGOaakcqWGlbWsdaWgaaWcbaGaemizaqgabeaakiabgUcaRiabcUfaBjabdcfaqnaaBaaaleaacqaIWaamaeqaaOGaeiyxa0Laey4kaSIaei4waSLaemitaW0aaSbaaSqaaiabicdaWaqabaGccqGGDbqxcqGGPaqkdaahaaWcbeqaaiabikdaYaaakiabgkHiTiabisda0iabcUfaBjabdcfaqnaaBaaaleaacqaIWaamaeqaaOGaeiyxa0Laei4waSLaemitaW0aaSbaaSqaaiabicdaWaqabaGccqGGDbqxaSqabaaakiaawIcacaGLPaaaaaa@7441@

Here, *F *is the fluorescence amplitude and *F*_0 _its value in the absence of ligand. Δ *F *is the normalized amplitude of the saturated quenching, [*PL*] is the concentration of liganded protein, [*P*_0_] and [*L*_0_] are the concentrations of the total protein and ligand, respectively. The protein concentration was determined from its 280 nm absorbance and pertains here to the monomeric unit (see below). Figure 1A shows the change of fluorescence amplitude as a function of added pyruvate. The solid line shows the best fit obtained using the above equation, yielding *K*_*d*_≈ 0.26 μM.

As described below, it appeared that the protein was in fact homodimeric and one may wonder whether any cooperativity is taking place between the two monomers. In fact, the satisfactory fit with a simple binding function excludes that significant cooperativity or anticooperativity be present. This is illustrated by the dashed line that was computed by simulating a small cooperativity (see legend) and clearly represents the upper limit that could accommodate the data in this respect. The titration experiments show that the binding occurs on a single, homogeneous site. To ascertain that this site corresponds to the monomeric unit (rather than, *e.g*., to the dimer), we also ran (data not shown) experiments at high protein concentrations (> *K*_d_). Under such conditions, the binding titration (or its initial part if [P_0_] is not very large with respect to *K*_d_) is essentially linear (all the added substrate is bound until saturation) and the concentration of binding sites is easily determined from the slope. The results confirmed that the number of binding sites was ~1 per monomer and that the protein was ~100% active for binding the substrate – in agreement with the crystal structure described in the following.

The *K*_*d *_values were determined for different structurally-related compounds as shown in Figure 1B, C and 1D. No binding was observed with α-ketoglutarate. In all cases the trend is the same as observed by Thomas *et al *[15]. The length of the aliphatic backbone chain clearly influences the affinity, a result that will be discussed later in the light of the structure. We next focus on the structural characterization of the interactions of TakP with 2-oxoacids, using pyruvate as a model substrate.

### A dimeric venus-flytrap with a swapped helix

We determined the crystal structure of TakP in its unliganded form and as a complex with pyruvate. The structure of the selenomethionine-labeled protein in its native form was first solved by the MAD method and then refined to 2.0 Å resolution with an *R*-factor of 17.9% (R_free _= 20.5% ; see Table 1). After a successful co-crystallization of TakP with pyruvate, the structure of the protein-substrate complex was solved by molecular replacement and refined to 1.4 Å resolution (*R *= 17.3%, *R*_free _= 18.4% ; Table 1 and Additional file 1 for an assessment of the quality of the electron density map). For the pyruvate complex all the residues fall in the favored region of the Ramachandran plot whereas in the native one, Trp215 and Val216 are outliers.

**Figure 2:**
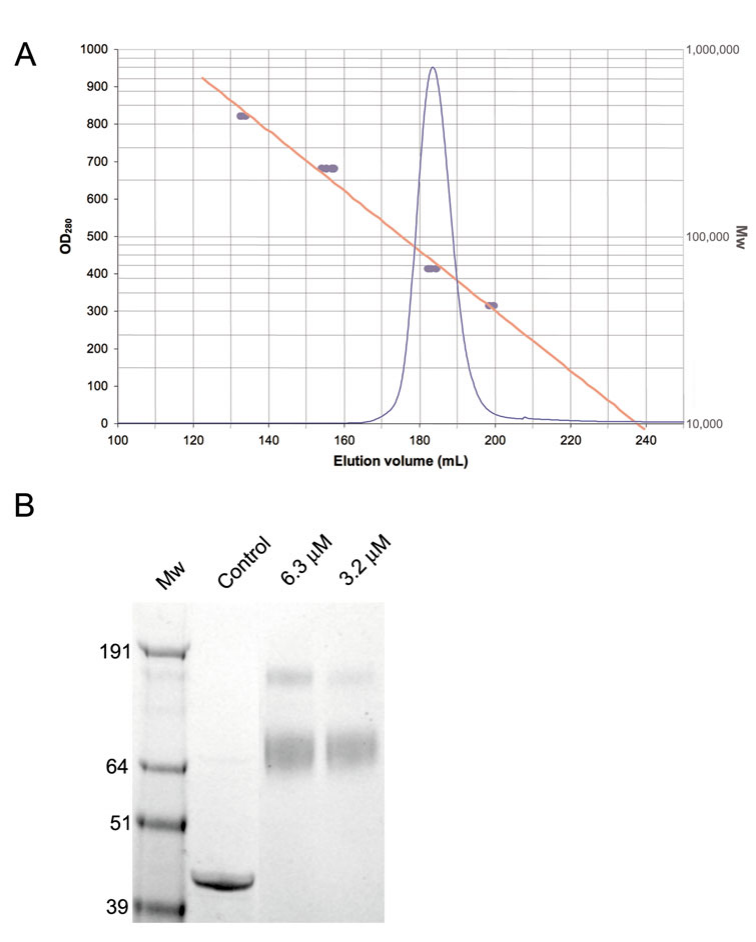
**Oligomeric state of TakP in solution**. **A**: Elution profile of a gel filtration experiment with TakP in 50 mM NaCl, 20 mM Tris HCl pH 8.0. A standard curve is superimposed and was calculated using the known molecular weight of four protein standards (blue circles). The theoretical molecular weight of TakP is 39 kDa. **B**: Denaturing gel electrophoresis before (Control) and after 3 hours incubation with the cross-linker glutaraldehyde (50 mM). Two concentrations of TakP were used as indicated. Each lane contains 2 μg of protein.

Unexpectedly, the resolved structures revealed a dimeric association of the protein in two different crystal forms, which is clearly too intricate to arise from the crystallization process. Because this quaternary structure is unusual for an ESR we confirmed the dimeric nature of TakP in solution by gel filtration and cross-linking experiments (Figure 2). In the cross-linking experiment, even at the lowest protein concentration tested (3.2 μM) the main band corresponds to the dimer. The monomeric form is not visible. Therefore, the dissociation constant of this dimer is at least ten times below this value (< 0.3 μM). Given the high concentration of proteins in the periplasmic space the dimeric nature of TakP *in vivo *is unambiguous. A tetrameric form of TakP is also visible, especially at higher concentration (6.3 μM) and may represent a real functional property of TakP to assemble as a multimer.

**Figure 3:**
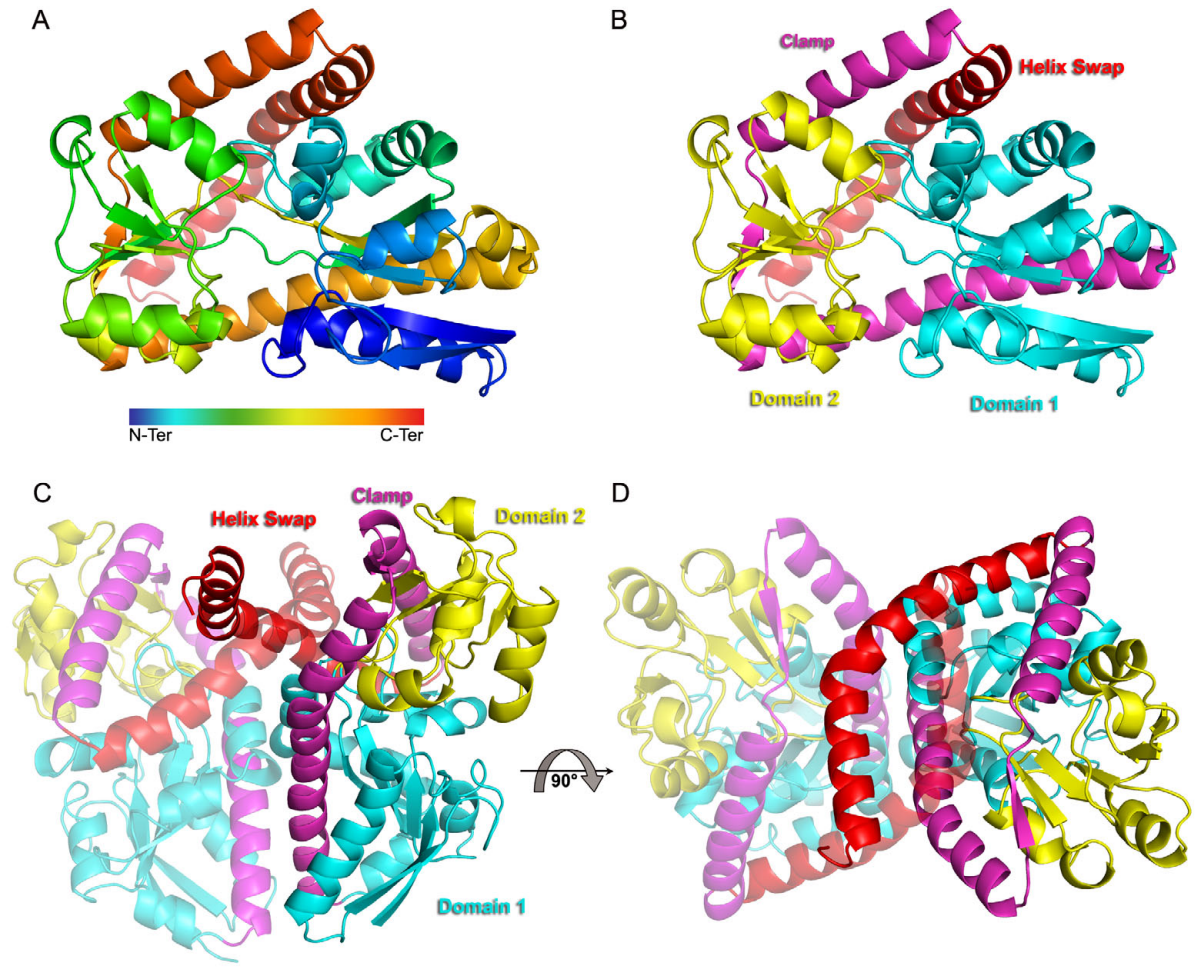
**Overall structure of TakP**. **A**: View of the TakP monomer colored as a ramp from blue to red from the N- to C-Terminus. **B**: View of the TakP monomer with colors according to the different structural domains. **C, D**: Two different orientations of the TakP dimer. Both monomers are colored as in B but one is slightly transparent and result in a paler coloring.

**Figure 4:**
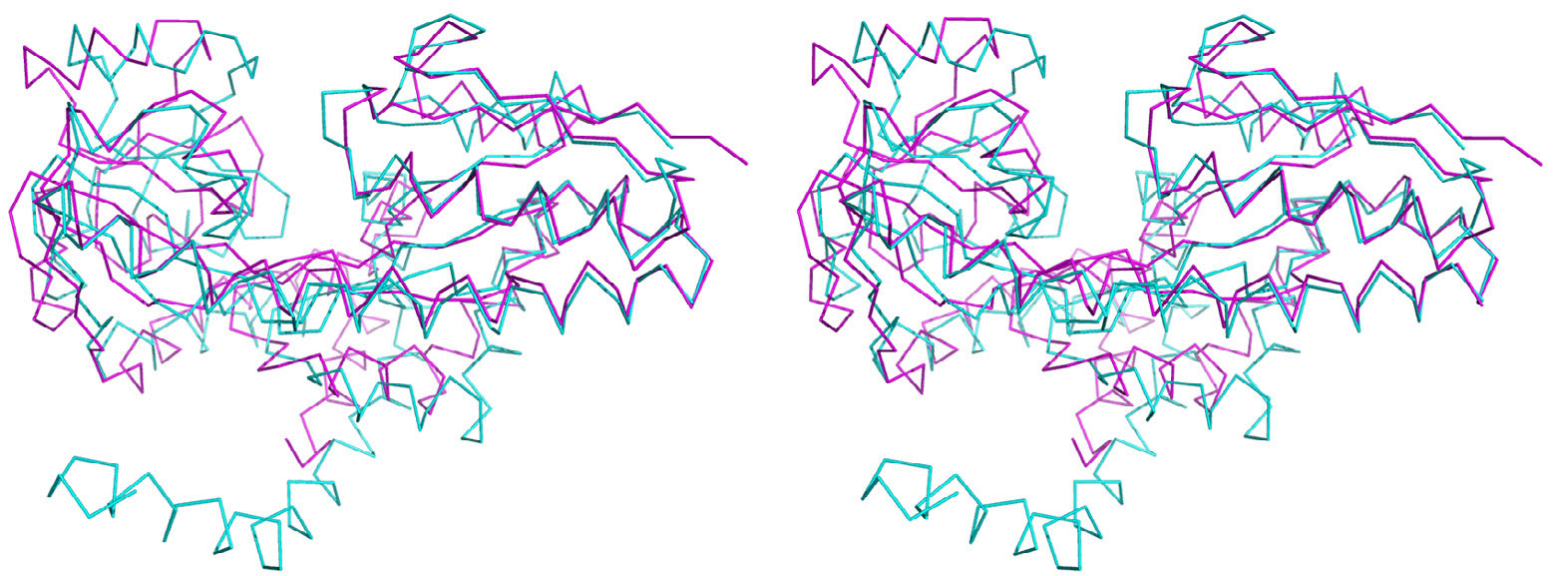
**Comparison of the TakP ad SiaP structure**. Stereo figure of a superimposition of the Cα positions of TakP (blue) and SiaP (magenta), both in their unliganded form.

Within a root mean square (r.m.s.) deviation below 0.3Å, the two monomers of the asymmetric unit appear essentially identical. We describe first the monomer's structure and then the dimeric features. This monomer appears subdivided into two globular domains, each with a 5-stranded β-sheet flanked by α-helices (Figure 3A). Domain I (from residue 32 to151 and 245 to 257) and domain II (154 to 242) are connected by two short segments (152–153 and 243–244), which play the role of a hinge allowing the open/closed transition. A structural homology search using the program DALI [17] revealed a large number of structures similar to TakP. Among them, the TakP monomer is structurally most similar to the glycine betaine-ESR (Z = 9.5 [18]), to the extracellular ligand-binding domain of the glutamate receptor (Z = 8.4 [19]) and to an ATP phosphoribosyltransferase (Z = 8.2 [20]). The two first structures were solved in the liganded form whereas the ATP phosphoribosyltransferase was solved in an open form. Thus, and despite very low sequence similarity, the TakP fold is characteristic of a type II ESR [2,3]. Recently, the crystal structure of SiaP was solved in the presence of a sialic acid analogue [13]. SiaP and TakP are both members of the DctP family. They share 20% sequence identity and, despite an overall fold conservation, their structures differ markedly with a r.m.s. deviation of 1.7 Å for the 167 best superimposed Cα positions, both structures in their unliganded form (Figure 4). Nevertheless, one secondary structural element seems to be unique to both TRAP ESRs compared with others: located after the C-terminal part of domain I, a "clamp" (from residue 258 to 325) surrounding both domains I and II is made of two long α-helices separated by a short β-strand parallel to the last strand of the β-sheet of domain II. Together with the dimerization, this clamp may limit the extent of the opening movement (see Figure 3C and Additional file 2).

**Figure 5:**
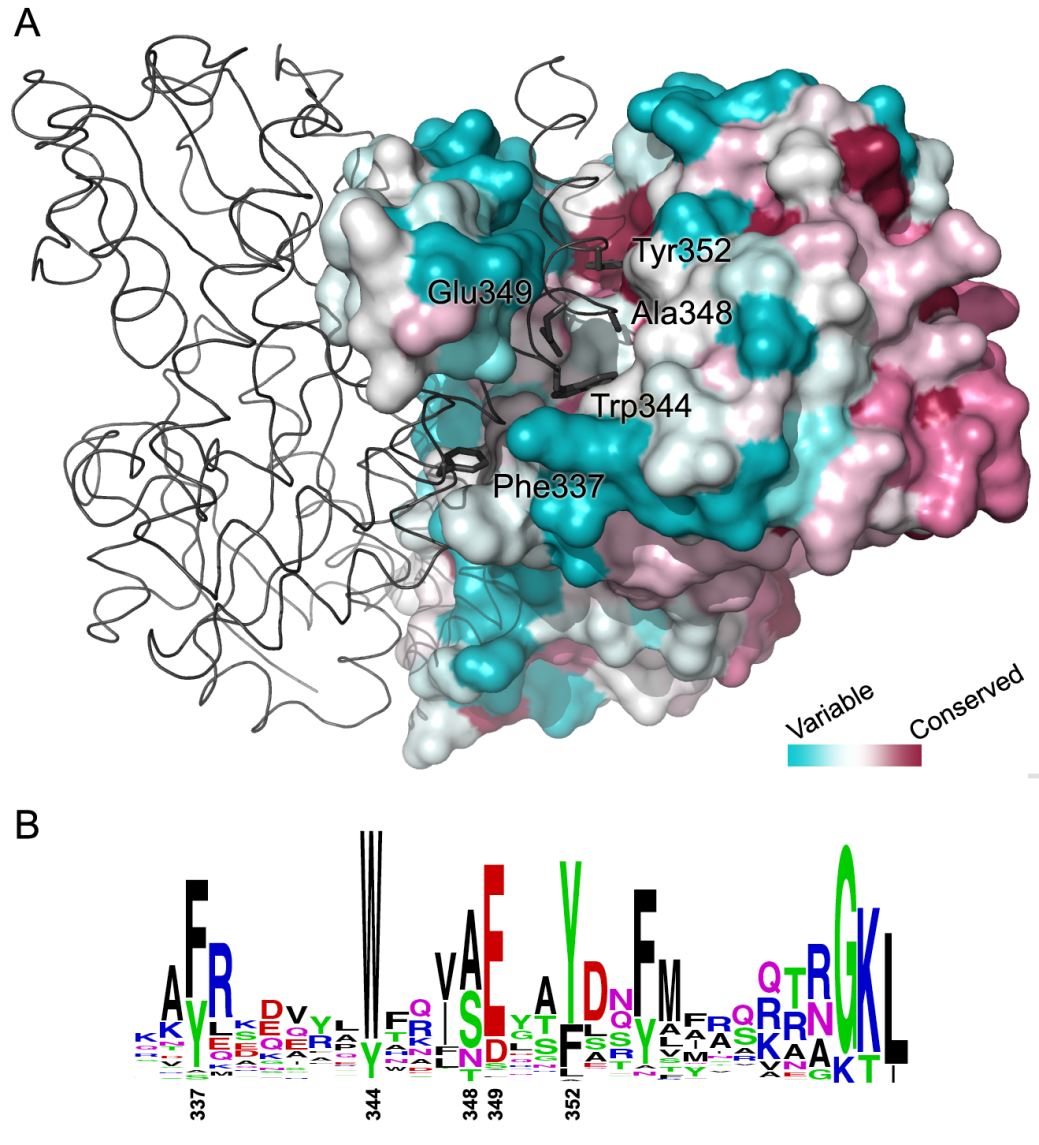
**A conserved dimeric interface**. **A: **The structure of one monomer is represented as a surface that is colored according to the sequence conservation pattern generated using 100 homologous sequences and as found by ConSurf [42]. **B: **Weblogo representation of the terminal swapped helix using the same set of sequences. In this representation the overall height of a stack indicates the sequence conservation at that position, while the height of symbols within the stack indicates the relative frequency of each amino acid at that position.

Another structural feature specific to TakP is a 55Å-long C-terminal kinked helix (from residue 326 to 365) extended away from its monomer and swapped with the equivalent helix from the adjacent monomer (Figure 3B). This unique secondary structure element is clearly essential for the dimerization of the protein. The terminal helix swapping appears critical for dimerization since it involves about 40 residues and generates numerous intermolecular contacts. The helix is amphipathic with the hydrophobic residues packed against the back of the adjacent monomer at the dimeric interface and the hydrophilic residues exposed to the solvent. In addition to the large total surface area (7180 Å^2^) buried in the complex, the dimer is stabilized by a total of 23 H-Bonds and two intermolecular salt bridges (detected using the program Proface [21]). The dimer formation brings two monomers in a back to back configuration with the conserved residues in the swapped helix of one monomer interacting with a conserved surface located on the back of the other monomer (Figure 5). The sequence conservation in multimeric proteins tends to be enhanced at the interacting interface [22]. This is indeed the case and clearly visible from Figure 5.

A conspicuous feature of the dimeric association in TakP is the presence of a water-filled channel buried at the dimer interface, which spans 30 Å from one ligand binding cavity to the other. As discussed later, we believe that substrate translocation through this connecting cavity may play a functional role.

### Co-recognition of a cation with pyruvate

**Figure 6:**
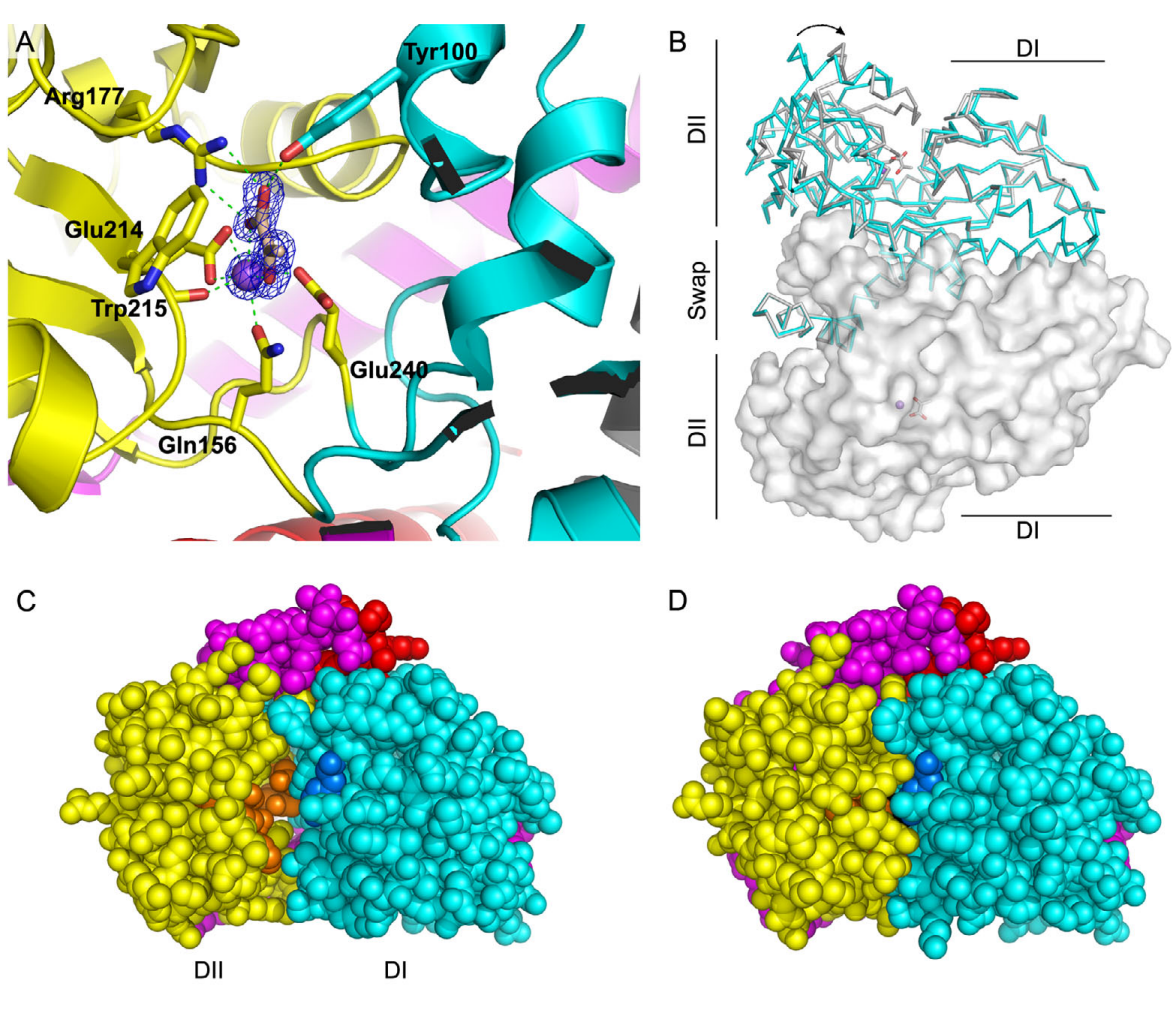
**Cation-pyruvate binding to TakP and structural changes upon ligand binding**. **A: **View of the binding region with the electron density omit map (omitting the pyruvate and the sodium ion from the calculation) together with a stick representation of the protein residues involved in the binding of the ligand. The sodium ion is represented as a purple sphere. The helix in red, visible at bottom of the panel, belongs to the other monomer. **B**: View of the overall changes induced by ligand binding. The unliganded protein is displayed as a cyan ribbon and the liganded protein is gray. For clarity, only the bound state of the other monomer is shown (gray surface). The distance between the two molecules of pyruvate from each monomer is 35Å. **C, D**: View of the interdomain closing (C, no ligand; D, liganded protein). TakP is represented in CPK, with the same color coding as in A, except for the residues interacting with sodium pyruvate, which are pictured dark blue and orange for residues belonging to Domain I and II, respectively. The pyruvate molecule (black, barely visible) is completely buried.

In the second crystal form obtained with the substrate-bound protein, the asymmetric unit contains one dimer with both monomers now found in the closed configuration. In the cleft between domains I and II of each monomer an extra electron density was unambiguously assigned to an ion-pyruvate complex (Figure 6A). The two solute binding sites in the dimer are located on opposite sides resulting in a distance between substrates of ~35 Å (Figure 6B).

In the complex structure, the acidic moiety of the pyruvate is involved in a salt bridge with Arg177 and one oxygen atom is H-bonded to Tyr100. The central feature for the binding of the pyruvate to the protein is the presence of a cation (Figure 6A). This cation has a bi-pyramidal coordination with a square base. The four equatorial positions are provided by the O3 of pyruvate, the main chain carbonyl oxygen of Trp215 and a bi-dentate and mono-dentate ligand provided by the side chains of Glu214 and Glu240, respectively. The apical positions are occupied on one side by an oxygen atom from the acidic moiety of pyruvate and, on the other side, by the Oδ1 of the side chain of Gln156. The measured distances between the cation and the six oxygen atoms range from 2.34 Å to 2.43 Å. These values are very close to the canonical distances expected for a magnesium or a sodium ion (R_Li _= 2.14 Å; R_Mg _= R_Na _= 2.46 Å ; R_Ca _= 2.66 Å ; R_K _= 2.77 Å [23,24]). Since only sodium salts were present in our crystallization conditions this cation can be safely identified as a sodium ion. Among the six residues involved in the recognition of sodium-pyruvate, only one (Tyr100) is provided by domain I, while the others belong to domain II or to the hinge (Figure 6A).

### Structural changes upon sodium pyruvate binding

Two kinds of conformational changes occur upon complex formation. First, there is a 14° rigid body rotation of domain II (Figure 6B; rotation was calculated using the DYNDOM program). Indeed, superimposition of the liganded and unliganded structures results in a r.m.s. deviation of 1.6Å for 334 Cα positions, a value that is an average of a high r.m.s. deviation measured for domain II (3.3Å for 89 Cα positions) and a rather low value for the rest of the molecule, comprising domain I, the clamp and the swapped helix (0.6Å for 245 Cα positions). The inter-domain closing is dominated by van der Waals contacts with only one hydrogen bond (Tyr99OH – Glu240Oε2) between domains I and II in the closed form. The dimerization interface is not modified by the open/closed transition, i.e. the intermolecular hydrogen bonds and salt bridges are conserved. Only one salt bridge (Glu340A-Lys289B) is specific to the closed conformation. Because of the absence of the symmetric salt bridge (Lys289A-Glu340B), the dimer appears slightly asymmetric.

The second conformational change associated with ligand binding corresponds to a small but significant structural rearrangement inside domain II, reflected by a r.m.s. deviation of 1.2 Å after superimposition of the 89 Cα positions of this domain in both structures. This rearrangement is mainly located in a loop-helix-loop region comprising residues 178 to 201, a portion of the structure that is not involved in the direct binding of sodium or of pyruvate. The movement of this region upon substrate binding locks the inter-domain closing by increasing the fit between both domains, which results in a ligand binding cavity completely shielded from the external solvent (Figure 6C and 6D). On the other hand, the internal water channel connecting the binding cavities is not affected by the open/closed transition.

## Discussion

Ligand binding kinetics determined for numerous ESRs from ABC transporters by stopped-flow fluorescence spectroscopy have revealed a single-step equilibrium binding process. These data suggest that the protein is stable in the open unliganded conformation, and that ligand binding triggers the closing of the globular domains. This model has been supported by crystallographic studies, which provided evidence for the existence of three different conformational states of ESRs: an open unliganded form, an open liganded form and a closed liganded form [3]. In the case of ESRs from the DtcP family, an uncommon kinetic behavior was reported [25], since the rate of substrate binding decreased when increasing its concentration. A model was proposed, involving a fourth ("closed unliganded") form of the protein. The binding would occur through the equilibrium between this predominant, non-binding, form and the open, binding configuration [25]. Our data show that the unliganded TakP was crystallized in the open configuration. This does not exclude the possibility that a more stable closed form might exist in solution, but it makes this hypothesis less likely.

Despite the very low sequence identity, TakP has been previously classified into family 7 of ESRs in the Pfam database. The high resolution crystal structure of TakP confirms an ESR overall fold. However, two additional secondary structure elements, a "clamp" and a helix swap, were not anticipated from primary sequence analysis. The topology and location of the clamp suggests a structural role in restricting the rigid body movement of domain II upon substrate binding. The restricted opening of TakP caused by the clamp appears when comparing the liganded and unliganded structures of ESRs. The two β-strands hinge allows a rather large rotation of domain II upon ligand binding (from 17° for the Nickel- to 56° for D-ribose- binding proteins; the average is 45°) whereas it is significantly smaller in TakP (14°) and below the average in SiaP (27°). The swapped helix also largely contributes to the limited opening of TakP as can be viewed in a movie provided as additional file 2. The requirement for a restricted movement upon solute binding might be important for the energetics of ligand binding. Since the residues responsible for the binding of the substrate belong almost exclusively to domain II, the proximity of the two domains in the apo state may facilitate the substrate-induced closing.

**Figure 7:**
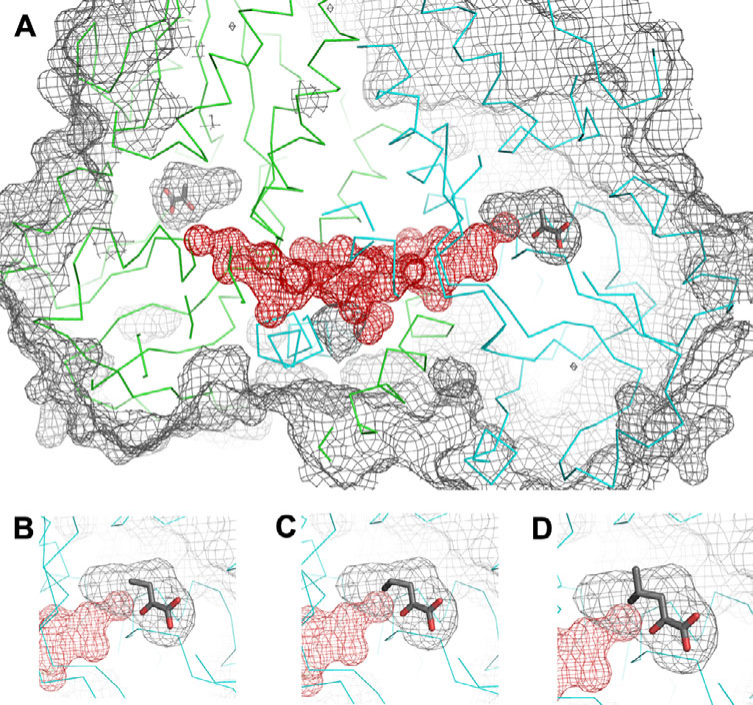
**View of the binding pockets and connecting channel**. **A**: a long water channel (red mesh) located at the interface of the two monomers opens onto both pyruvate binding sites. **B, C, D**: Models showing how various ligands may fit into the binding pocket : oxobutyrate (B), oxovalerate (C) and 4-methyl-2 oxovalerate (D). The *K*_*d*_'s found for these molecules is indicated in Figure 1.

The natural ligand(s) of TakP is still unknown [15]. It is not necessarily pyruvate, but it should include a pyruvate motif. We made a structural model for each of the ligand molecules that were tested by fluorescence quenching. The ligand's three-dimensional structure was modeled as the lowest energy structure using the 3D utility from ChemDraw. The molecular cavity in TakP was visualized with the pyruvate moiety of the modeled ligand superimposed on the pyruvate structure. As a result, it clearly appears that the increased length of carbon chains from oxobutyrate (Figure 7B) and oxovalerate (Figure 7C) fit better the binding pocket than pyruvate (Figure 7A), explaining why an increase in the chain length of the aliphatic backbone increased the ligand-binding affinity (Figure 1). An even longer chain elongated by one or two carbon atoms is predicted to have a higher affinity because there is still enough space to accommodate it. In addition, the reason why a branching carbon at position 4 (4-methyl-2 oxovalerate) increases the *K*_*d *_is obvious from Figure 7D, where this position is clearly outside of the cavity. Since the ligand-binding cavity is rather hydrophobic and delineated by the side chains of Y99, F40, V97, V245 A212, A213, P243, T153 and I47, only ligands with a carbon chain of similar length are expected while similar compounds with a charged or a polar moiety should be poor ligands, a behavior observed in the case of α-ketoglutarate.

As revealed by the present work, a sodium ion plays a key role for the binding of the 2-oxo acids to the protein. Since TRAP transporters utilize the transmembrane electrochemical Na^+ ^or H^+ ^gradient as the driving force for solute import, it is tempting to believe that the concerted recruitment by the ESR of the substrate group with a cation is a first step in the coupled transport of both partners [26].

The presence of a swapped helix in TakP was an original and unanticipated feature. The 40 swapped residues significantly contribute to the total surface that is buried in the dimer, therefore playing a clear role in the dimer formation. About a hundred proteins were found to be homologue to TakP and they all have in common the long C-terminal extension corresponding to the swapped helix. Because of its importance in dimerization this helix may be a marker of a novel family of dimeric ESRs. This dimeric nature of TakP is intriguing because ESRs, especially when associated with an ABC transporter, are believed to be monomeric. To our knowledge, there is one report of dimer formation, based on biochemical evidence [27]. However, sequencing has now revealed the presence of ABC transmembrane dimeric subunits directly fused to one or two ESRs, which implies that two or four binding sites are present per functional translocation unit [28]. Multimerization of ESRs that are not associated to ABC transport systems is exceptional and then appears linked to a particular function: activation of transport in glutamate receptors (a close structural homolog of TakP found by DALI) and activation/repression of DNA translation by DNA-repressor of the LacR family. In lactoferrin and transferrin, two ESRs are fused into a single protein and are therefore associated as a "forced dimer" [29]. The role associated with the dimerization of TakP remains to be investigated. Our binding data exclude any significant cooperativity between the two sites, which is consistent with the structural results showing no significant modification of the monomer/monomer interfacial regions upon binding the ligand. A possible role for the dimerization of TakP is proposed below.

**Figure 8:**
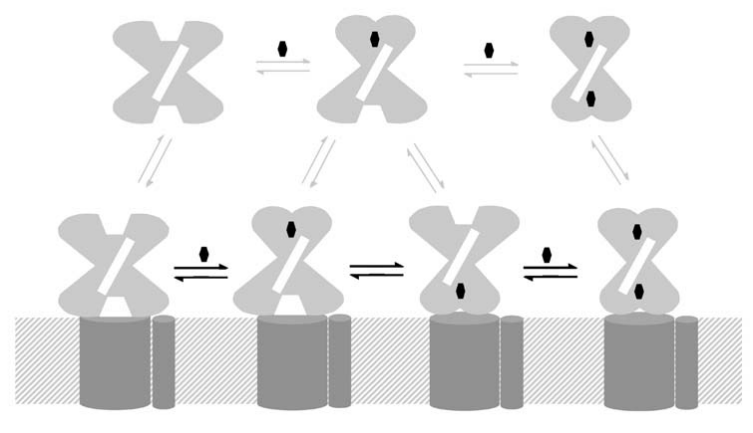
**Model of interaction of the TakP dimer with its membrane partners TakQ and TakM**. In this model, a membrane-bound pathway is hypothesized (heavy arrows). The channel at the dimer interface would facilitates the passage of the substrate from the solvent-exposed monomer to the other monomer that interacts with a membrane subunit. This model does not exclude the possibility that binding of the loaded ESR may happen (light arrows).

The molecular interactions between the solute-ESR complex and the multisubunit transporter are still unclear. One view is that, as a result of the conformational change associated with solute binding, the ESR-substrate complex becomes competent to interact with the membrane subunits of the ABC transporter, initiating the transport cycle [30]. On the other hand, it was shown that, at least in one subfamily of ABC transporters, both the unliganded and ligand bound ESR could interact with equal affinity with the translocating membrane protein [31]. Some authors proposed that, in addition to the recruitment of the transported ligand, the ESR plays the role of a plug that prevents the escape of the ligand on the wrong site of the membrane [28,32,33]. In line with these views, the present structure of a dimeric ESR suggests a possible model of interaction with the membrane partners (the membrane bound pathway pictured in Figure 8). In this model, the dimeric ESR would stay more or less permanently bound to the membrane protein. This would break the functional symmetry in the homo-dimeric TakP protein: the external binding site would be used for substrate and ion uptake from the solvent, and the connecting channel at the dimeric interface would facilitate the transport to the site on the membrane bound monomer. This model is consistent with the recent finding that one ore two pairs of ESRs are sometimes directly fused to the membrane protein [28,34]. Definitely, further work is needed to probe this model, whether in the context of an ABC transporter or, like in the present case, of a TRAP transporter.

## Conclusion

The X-ray structures of TakP solved in the presence and in the absence of substrate reveal the structural determinants responsible for the binding of α-keto acids. The requirement of a cation for the binding of the ligand is suggestive of a role in the energization of the transport. Moreover, an unexpected helix-swapped dimer is observed in two different crystal forms. Although this quaternary structure does not generate a cooperative binding of the substrate, the connecting channel at the dimeric interface could be involved in the translocation process.

## Methods

### Cloning

Vector pET101/D-TOPO (Invitrogen) containing a T7 promoter, a C-Terminal His_6 _tag, and V5 epitope was used for cloning and expression. Two synthetic oligonucleotide primers were designed in order to amplify the *TakP *gene from *Rhodobacter sphaeroides 2.4.1 *genomic DNA. The 1099 bp fragment obtained after amplification was electrophoretically separated on a 1% SeaKem GTG-agarose gel and extracted prior to cloning into pET101/D-TOPO.

### Bacterial expression and protein purification

The recombinant protein was overexpressed in *E. coli *BL21 strain. Cells were allowed to grow in LB (Lenox Broth) medium containing 100 mg/ml ampicilline to an OD_600 _of 0.7 before inducing expression with 1 mM Isopropyl-β-D-thiogalactopyranoside (IPTG) followed by 20 hours incubation at 30°C. The cells were pelleted, resuspended in buffer A containing 50 mM phosphate pH 8.0, 450 mM NaCl, and disrupted with a French Press at 7 Mpa. The resulting soluble fraction was loaded on a Nickel charged column (HisTrap column, Amersham) and the protein was eluted by an imidazole step gradient (40 mM wash and 200 mM elution). The recombinant selenomethionine labeled protein was overexpressed in *E. coli *after growth in a 1 L of methionine-minus medium from Molecular Dimensions, complemented with 40 mg of L-selenomethionine (SeMet), L-lysine, L-threonine and L-phenylalanine as described [35]. Purification of the SeMet TakP was performed as previously, except the addition of 1 mM Tris(2-carboxyethyl)phosphine (TCEP) in the different buffers.

### Cross-linking and gel filtration chromatography

The cross-linking experiment was performed by incubating 3.2 and 6.3 μM TakP with 50 mM glutaraldehyde, in 20 mM Na_2_HPO_4_/KH_2_PO_4 _pH 7.8, at 18°C for 3 hours. The control reaction contained no cross-linker. The denaturing gel electrophoresis was done with a 10% Nu-PAGE gel (Invitrogen), in MOPS buffer. The gel filtration chromatography was done using a Superdex 200 26/60 column (Amersham Biosciences) equilibrated with 20 mM Tris HCl (pH 8.0), 50 mM NaCl. The column was previously size calibrated using commercial gel filtration standards (Amersham Biosciences).

### Crystallization

For crystallization, the native protein was exchanged into buffer B (50 mM Tris HCl pH 8.5, 180 mM NaCl) and concentrated up to 15 mg.ml^-1^. All the crystallization experiments were carried out at 293 K using the hanging drop vapor diffusion technique. Crystals of the unliganded protein were obtained by mixing 2 μl of protein with 2 μl of a reservoir solution (500 μl) containing 100 mM sodium citrate (pH 5.8–6.0) and 1.4–1.6 M ammonium sulfate. In these conditions, three different crystal morphologies were obtained: cylindrical rods, large hexagonal crystals and, more rarely, a large stacking of finely faceted plate crystals. Only crystals extracted from this bundles diffract x-rays. They grew in about five days and belong to space group *P*2_1 _(Table 1). In order to get a structure of a pyruvate complex we re-screened various co-crystallization conditions with 30 mM pyruvate. Crystals were obtained in approximately the same conditions as for the native, i.e. by mixing 2 μl of the native protein with 2 μl of a reservoir solution containing 100 mM sodium citrate (pH 5.4), 1.4 M ammonium sulfate and 30 mM sodium pyruvate. Crystals grew in about 1 week and correspond to a different crystal form (Table 1).

### Data collection and phasing

Native and SeMet crystals were cooled to 100 K after soaking for several minutes in a cryobuffer containing 30% glycerol whereas paraffin oil was used as cryoprotectant for the pyruvate-TakP crystals. Diffraction data on both the Se-Met labeled protein and the protein pyruvate complex crystals were collected at beamline ID23 (ESRF, France). The data sets were processed with MOSFLM [36] and subsequent data reduction was carried out using the CCP4 suite [37].

### Structures determination and refinement

The structure of SeMet TakP was solved by the MAD method using data collected at three wavelengths. Fourty selenium positions were found from the MAD data by the program SOLVE [38]. The four operators relating the four molecules in the asymmetric unit were found by the program and approximately 75% of the molecule was subsequently automatically built. Several rounds of iterative model building and refinement were performed using the programs COOT [39] and REFMAC [40]. The final model contains four molecules (from residue 28 or 32 to 365), 760 solvent molecules and four molecules of glycerol. The structure of the protein in the presence of pyruvate was subsequently solved by molecular replacement using the unliganded structure as a search domain. It quickly appeared that domain II had a different orientation than in the native structure. The prime and switch option of SOLVE was used to increase the quality of the electron density map and the resulting map was then easily interpretable. After several cycles of model building and refinement with REFMAC, the final model contained 2 molecules of TakP, 2 sodium pyruvate complexes and 470 solvent molecules. In both structures, the structural parameters were consistently better than or within the average for structures at comparable resolution [41].

### Fluorescence spectroscopy

Fluorescence spectroscopy was performed using a Varian Cary Eclipse spectrofluorimeter with an excitation wavelength of 290 nm. The emission spectra were recorded between 300 and 500 nm. Ligand binding was determined from the partial quenching of this fluorescence emission. To gain a more accurate determination of the dissociation constant, the protein concentration was lowered down to 50 nM (in 50 mM Tris/HCl, pH 7) and the amplitude of the fluorescence emission was computed from an integration of the emission over the 315 nm to 360 nm range.

## Authors' contributions

P.A., M.S. and D.P. designed the research; S.G., B.P., B.A., M.S. carried out the molecular genetic study, SG, P.A. and D.P. purified and crystallized the protein, collected X-ray data and solved the structures, J.L. analyzed the fluorescence data, and P.A., J.L. and D.P. wrote the paper. All authors read and approved the final manuscript.

## Supplementary Material

Additional file 1 **Quality of the electron density map at the dimeric interface. **Each monomer is based on a single color (green or blue). The electron density map corresponds to the final refined 2Fo-Fc map and is colored according to the monomer to which it belongs, except for water molecules that are here colored in gray. Click here for file

Additional file 2 **A movie depicting the transition between the open – closed transition in TakP. **The coloring scheme in this movie is according to the domains as they are defined in Figure 3. It is apparent that the movement of domain 2 on one monomer is somehow hindered by the swapped helix of the other monomer. Click here for file

## Figures and Tables

**Table 1:** Summary of crystal parameters, data collection and refinement statistics

**Data collection**	Unbound Selenomethionine (*P*2_1_)			Pyruvate (*C*2)
Wavelength, Å ^a^	0.97960 (i)	0.97940 (p)	0.97565 (r)	0.97565
Resolution range, Å	68-1.7	68-1.8	68-2.0	48-1.4
Cell parameters:				
a	104.61			118.02
b	63.94			78.06
c	127.95			95.14
β	106.64°			124.98°
No. of measured reflections	581707	506662	374656	500384
No. of unique reflections	175148	149602	109442	137318
*R*_sym_^*b*,*c*^	8.7 (46.2)	8.0 (25.3)	7.4 (18.3)	7.7 (49.1)
I/σI^*c*^	12.3 (2.2)	13.1 (3.6)	13.5 (5.3)	15 (3.1)
Completeness, %^*b*^	98.5 (95.5)	99.5 (98.2)	99.6 (98.6)	99 (97.9)
Sites (n)	40			
**Refinement**				
Resolution range, Å	68-1.7			48-1.4
R_cryst _/R_free_	17.9/20.5			17.3/18.4
No. of non hydrogen atoms:				
Total	11298			5795
Protein	1054			5304
Glycerol	24			
Pyruvate	-			12
Ion	-			2
Water	731			477
Average B-factors, Å ^2 ^:				
Main-chain	16.1			9.6
Side-chain	18.2			11.3
Glycerol	35.8			-
Pyruvate	-			9.2
Ion	-			4.4
Water	24.8			19.6
RMSD bonds, Å	0.012			0.007
RMSD angles, °	1.30			1.14
PDB code	2HZK			2HZL

^*a *^(i), (p) and (r) denote respectively inflection, peak and remote data sets.

^*b *^*R*_sym _= Σ_h_Σ_i_|*I*_i_(**h**) - <*I*(**h**) >/Σ_h_Σ_i_*I*_i_(**h**)

^*c *^Number in parenthesis refer to the last 0.1Å shell
