# Supplementary figures and images for: Crystal structures of an Extracytoplasmic Solute Receptor from a TRAP transporter in its open and closed forms reveal a helix-swapped dimer requiring a cation for α-keto acid binding
Source: BMC Struct Biol. 2007 Mar 15;7:11. doi: 10.1186/1472-6807-7-11 (PMC1839085; doi:10.1186/1472-6807-7-11)

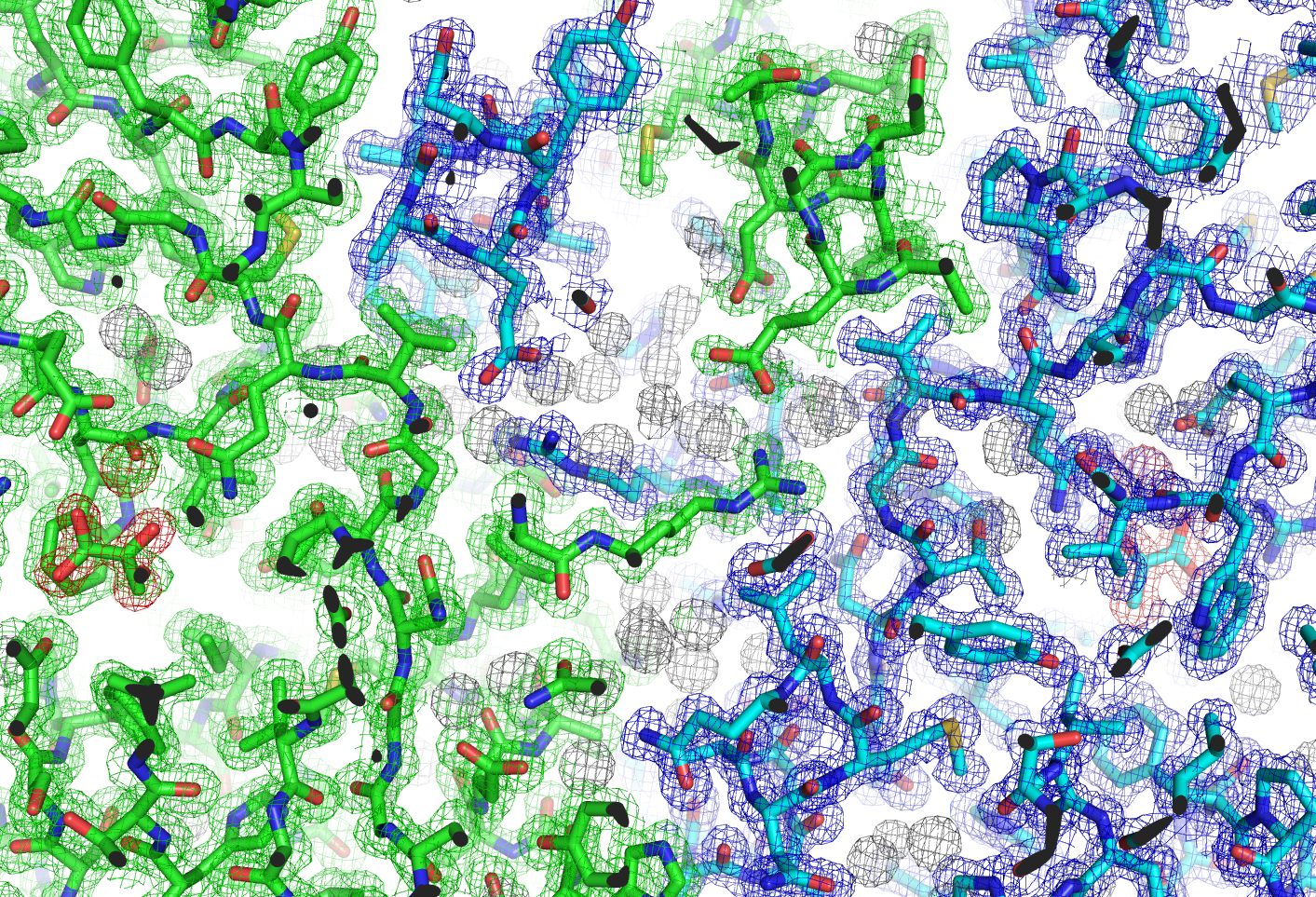

Supplement: Additional file 1 — Quality of the electron density map at the dimeric interface. Each monomer is based on a single color (green or blue). The electron density map corresponds to the final refined 2Fo-Fc map and is colored according to the monomer to which it belongs, except for water molecules that are here colored in gray. [file 1472-6807-7-11-S1.tiff]
